# Omics technologies provide new insights into the molecular physiopathology of equine osteochondrosis

**DOI:** 10.1186/1471-2164-15-947

**Published:** 2014-10-31

**Authors:** Clémence Desjardin, Julie Riviere, Anne Vaiman, Caroline Morgenthaler, Mathieu Diribarne, Michel Zivy, Céline Robert, Laurence Le Moyec, Laurence Wimel, Olivier Lepage, Claire Jacques, Edmond Cribiu, Laurent Schibler

**Affiliations:** INRA, UMR1313, Biologie Intégrative et Génétique Animale, Jouy-en-Josas, France; CNRS, PAPPSO, UMR 0320/UMR8120 Génétique Végétale, Gif-sur-Yvette, France; INSERM, U902 UBIAE, Université d’Evry Val d’Essonne, Evry, France; Station expérimentale des Haras Nationaux, IFCE, Chamberet, France; VetAgro Sup, GREMERES-ICE, Campus Vétérinaire, Marcy l’Etoile, France; UR4, Pierre and Marie Curie University, Paris, France; Ecole Nationale Vétérinaire d’Alfort, Université Paris Est, Maisons-Alfort, France; UNCEIA, Maison Nationale des Eleveurs, 149 rue de Bercy, 75595 Paris, Cedex 12, France

**Keywords:** Equine osteochondrosis, Cartilage, Subchondral bone, Proteomics, Metabolomics, μCT

## Abstract

**Background:**

Osteochondrosis (OC(D)) is a juvenile osteo-articular disorder affecting several mammalian species. In horses, OC(D) is considered as a multifactorial disease and has been described as a focal disruption of endochondral ossification leading to the development of osteoarticular lesions. Nevertheless, OC(D) physiopathology is poorly understood. Affected horses may present joint swelling, stiffness and lameness. Thus, OC(D) is a major concern for the equine industry. Our study was designed as an integrative approach using omics technologies for the identification of constitutive defects in epiphyseal cartilage and/or subchondral bone associated with the development of primary lesions to further understand OC(D) pathology. This study compared samples from non-affected joints (hence lesion-free) from OC(D)-affected foals (n = 5, considered predisposed samples) with samples from OC-free foals (n = 5) considered as control samples. Consequently, results are not confounded by changes associated with the evolution of the lesion, but focus on altered constitutive molecular mechanisms. Comparative proteomics and micro computed tomography analyses were performed on predisposed and OC-free bone and cartilage samples. Metabolomics was also performed on synovial fluid from OC-free, OC(D)-affected and predisposed joints.

**Results:**

Two lesion subtypes were identified: OCD (lesion with fragment) and OC (osteochondral defects). Modulated proteins were identified using omics technologies (2-DE proteomics) in cartilage and bone from affected foals compare to OC-free foals. These were associated with cellular processes including cell cycle, energy production, cell signaling and adhesion as well as tissue-specific processes such as chondrocyte maturation, extracellular matrix and mineral metabolism. Of these, five had already been identified in synovial fluid of OC-affected foals: ACTG1 (actin, gamma 1), albumin, haptoglobin, FBG (fibrinogen beta chain) and C4BPA (complement component 4 binding protein, alpha).

**Conclusion:**

This study suggests that OCD lesions may result from a cartilage defect whereas OC lesions may be triggered by both bone and cartilage defects, suggesting that different molecular mechanisms responsible for the equine osteochondrosis lesion subtypes and predisposition could be due to a defect in both bone and cartilage. This study will contribute to refining the definition of OC(D) lesions and may improve diagnosis and development of therapies for horses and other species, including humans.

**Electronic supplementary material:**

The online version of this article (doi:10.1186/1471-2164-15-947) contains supplementary material, which is available to authorized users.

## Background

Osteochondrosis (OC(D)) has been described as a focal failure or disruption of endochondral ossification [1] that occurs in young, growing individuals. This condition affects 10 to 30% of the equine population as well as other animal species such as pigs, poultry and dogs and also humans. Currently, the original term *osteochondrosis* (OC) is used for the forms without loose fragments or overt signs of inflammation, including subchondral bone cysts. O*steochondritis dissecans* (OCD) is used to refer to presentations where osteochondral fragments separate and become loose intra-articular bodies (joint mice), leading to further damage such as synovitis and pain accompanied by varying degrees of lameness [2]. In its early subclinical stages, where focal areas of cartilage necrosis are confined to the epiphyseal cartilage, the condition has been referred to as dyschondroplasia [3], or more recently *osteochondrosis latens*, whereas the presence of retained cores of cartilage and focal areas of cartilage necrosis visible on macroscopic and radiographic examination is designated as *osteochondrosis manifesta* [4].

In horses, a multifactorial origin is commonly accepted, including environmental factors (dietary imbalance and biomechanical factors), physiological factors (growth, conformation and hormonal imbalance) and genetics [5–7]. Despite substantial research efforts made since the early 1990s, including gene mapping studies (for review see [8]), OC(D) pathogenesis remains unclear [9], probably due to the confusion regarding disease definition and the lack of precise data about mechanisms of primary lesion formation [4]. In this respect two main hypotheses have been put forward to explain the origin of primary lesions: the vascular and the dyschondroplastic hypotheses. In the first one, a local defect in cartilage canal blood supply, in a certain age window, may lead to focal ischemic necrosis of growth cartilage and abnormal ossification [4, 10, 11]. In the second hypothesis, primary lesions result from a local failure of endochondral ossification [12]. A classification has been proposed recently, based on anatomical and functional aetiopathogenesis of lesion [13]. Histological studies have made it possible to grossly divide primary lesions into two main groups [3, 12, 14]: 1) lesions with an accumulation of chondrocytes apparently arrested in the pre-hypertrophic stage and associated with necrotic areas, and 2) lesions characterized by an alteration of mineralized matrix, necrotic areas, and increased type VI collagen immunoreactivity as well as cartilage cores retained in the subchondral bone [15]. Taken together, these features suggest a disruption of the endochondral ossification process at the chondro–osseous junction that impairs cartilage replacement by bone tissue as the ossification front advances with time [15]. In both hypotheses, shearing biomechanical forces are thought to drive the development of dissecting lesions and biomechanical influences may explain the local disruption of cartilage canals as well as the existence of predisposition sites [9, 11, 16]. The picture is also complicated due to the highly dynamic character of OC in young foals. Indeed, even large lesions may heal spontaneously without leading to any pathology, probably due to the high metabolic activity of juvenile cartilage that enables growth and facilitates repair of the tissue [9, 17].

There is clearly a need for a better understanding of OC(D) molecular physiopathology in order to improve lesion classification and increase positional cloning efficiency [9]. The aim of our study was to identify constitutive defects and altered biological processes in epiphyseal cartilage and/or subchondral bone associated with the development of primary lesions. In agreement with what is currently known, we considered that, in all joints, different molecular mechanisms are responsible for the development of the different forms of OC(D). These mechanisms may result in differing susceptibility to specific biomechanical forces, leading to a heterogeneous pathological profile. Therefore, a novel experimental approach was taken for the study of this pathology, in order to compare the proteome of epiphyseal cartilage and subchondral bone sampled from lesion-free joints of 10 month OC-free and OC(D)-affected foals. Consequently, results are not confounded by changes associated with the lesion development, disease progression or healing process.

## Methods

### Animal Care Committee

The experimental protocol number 0964 was reviewed and approved by the Animal Care Committee of VetAgro Sup (France), which abides by the requirements of Directive 86/609 of the European Community Council.

### Animals and inclusion criteria

The study was based on 33 Anglo-Arabian foals obtained from a unique stallion known to have OC-affected foals in his progeny. None of the dams were diagnosed as OC(D)-affected based on clinical examination. To reduce genetic and environmental variability, foals were bred at the experimental station of the French National Stud (Station IFCE de Chamberet) and mare-foal pairs were managed by rotational grazing until the end of the grass season. The osteo-articular status and identification of OC(D) lesions of each foal was determined between six to seven months of age based on current knowledge of lesion development and progression [17]. Clinical examination of the foals included a qualitative visual assessment of lameness, trauma, skin anomalies and joint effusion. Radiographic examination was performed for metacarpo and metatarsophalangeal joints as well as tarsocrural and femoro patellar joints (12 views) and X-rays were evaluated for the presence of OC(D) lesions. Additional X-rays were taken to confirm the presence or absence of lesions in the case of irregularities or anomalies. Tarsocrural joints were considered as affected if they had radiological evidence of OC(D) at any predisposition site (i.e. the intermediate ridge of the distal tibia, medial or lateral trochlear ridge of talus, and medial malleolus). Femoro patellar joints were considered OC(D)-affected if radiological anomalies were observed on the lateral or medial trochlear ridge of the femur. Foals were considered OC-free if they did not show any sign of trauma, lameness or joint effusion and their radiographic exam did not reveal any sign of OC(D) lesions on any limb joint.

Among the 33 foals, 12 were diagnosed as OC(D)-affected and 5 were selected for the study. Foals with signs of external trauma were excluded. Selected foals were chosen based on the affected joint and to balance groups in terms of gender and age. These foals (OC1-OC5) showed at least one lesion in tarsocrural joints (see Additional file 1: Table S3). These foals did not present any signs of lameness. Foals OC3, OC4 and OC5 were bilaterally affected, whereas foals OC1 and OC2 were only affected in the right joint. Noteworthy, no sign of OC(D) was observed in their femoro patellar joints. Five foals considered OC-free (OF1-OF5) were selected, based on their soundness at clinical examination combined with the absence of radiographic abnormalities on any of the 12 views.

**Figure 1:**
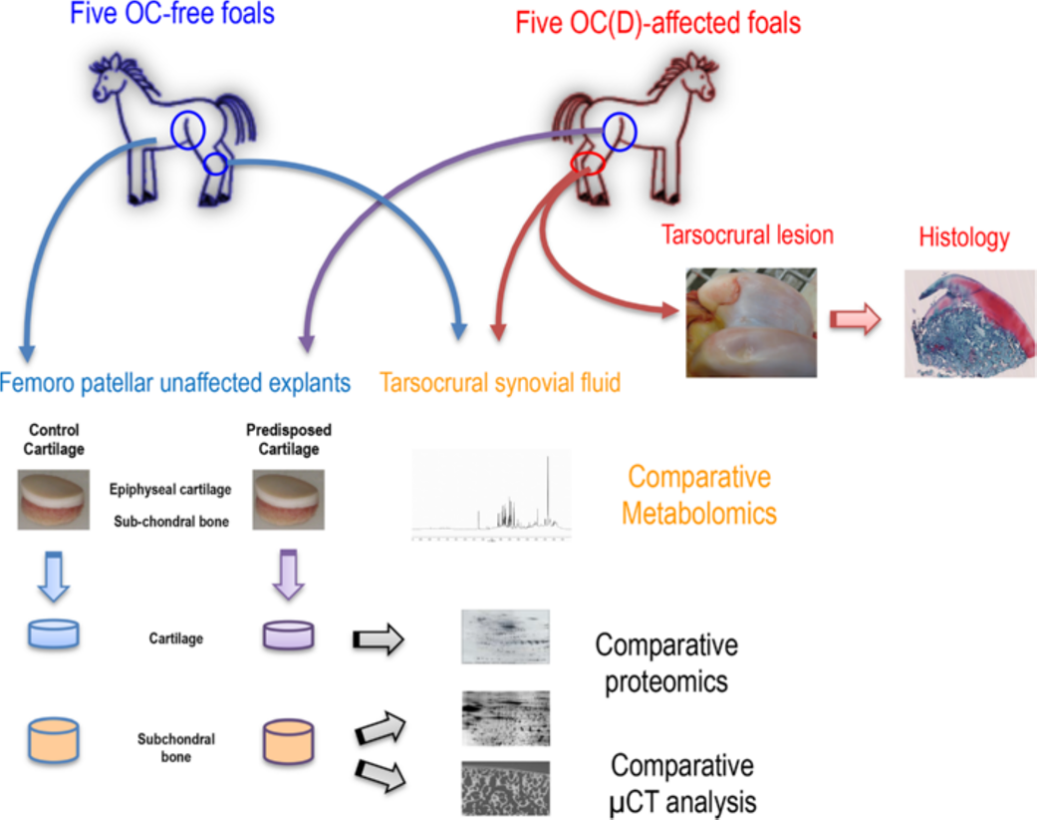
**Study design.** Ten foals were allocated to two groups based on clinical and radiographic assessment: OC-free (n = 5) and OC-affected (n = 5). OC foals presented at least one talocrural lesion whereas femoropatellar joints were normal. Lesions were sampled and classified histologically. Explants comprising both cartilage and 5 mm of subchondral bone were harvested on femoropatellar joints and considered as healthy samples if retrieved from OC-free foals and as predisposed samples if retrieved from OC-affected foals. Samples were used for comparative proteomics and micro computed tomography (μCT). Tibiotarsal joint synovial fluids were also collected from OC-free and OC(D)-affected foals (affected limb and contralateral non-affected limb for foals non-bilaterally affected) and used for comparative metabolomics. Blue arrows correspond to OC-free samples, purple arrows to predisposed samples and red arrows to OC(D)-affected samples.

Foals were euthanized at 10 months by lethal intravenous injection of T61 (Embutramide), a sedative drug used for euthanasia in veterinary medicine. The presence/absence of OC(D) lesions was macroscopically confirmed during necropsy by inspection of all limb joints, except for the coxofemoral and coffin joints (ie presence/absence of irregularities or abnormalities at the articular cartilage surface). A hammer and a-12-mm punch were used to collect cartilage and subchondral bone cylindrical explants from the femoral *trochlea* of OC-free and OC(D)-affected horses. As mentioned earlier all femoropatellar joints showed no gross or radiographic evidence of disease. These samples were considered as predisposed samples if retrieved from the affected group (OC1-5) and as control samples if retrieved from the OC-free foals (OF1-5). Three consecutives punches were taken in the middle of the trochlea (one for proteomics, one for μCt and one for histology) (Figure 1). All comparisons performed in our study involved punches collected at the same location (see Picture P1 in the Additional file 1).

### Histological analysis and immunochemistry

Osteochondral lesions for histological analysis were cut in the sagittal plane to include five mm of subchondral bone and fixed for 18 hours in a solution of 4% paraformaldehyde (PFA), decalcified for one month in 20 ml DC3 solution and embedded in paraffin. 5-μm sections were stained with hematoxylin-eosin-saffron (HES) and safranin O-Light Green (LGS).

### Proteomics

Cartilage and bone were carefully separated using a cutter and a hammer and immediately placed in liquid nitrogen. Frozen cartilage (200 mg) and bone (150 mg) samples were crushed in liquid nitrogen using a hammer and a stainless steel mortar and pestle and then ground to a fine powder in liquid nitrogen using a porcelain mortar and pestle. Protein extraction and quantification(2-D Quant Kit, Sigma Life Science), two dimensional electrophoresis (2-DE) and spot identification were then performed as previously described [18]. Spot volume normalization was performed using the Progenesis “Total Volume Ratio” option. Descriptive statistics were done using R scripts and the ade4 package. Principal component analysis (dudi.pca) and between-class correspondence analysis (dudi.bca) were performed to investigate differences between OC-free and OC(D)-affected horses as well as heterogeneity between affected samples. Spots having inertia above the third quartile and showing at least a 50% change in abundance between OC(D)-affected foals and OC-free were considered as associated with the pathology. A hierarchical clustering (Pearson correlation) was also performed based on a subset of differentially expressed spots (*t*-test, adjusted p-value <0.2).

### Comparative micro computed tomography (μCT)

Cylindrical cartilage and subchondral bone explants were obtained from undamaged femoro patellar joints of both OC-free and OC(D)-affected foals using a 12-mm punch. Samples were stored in 70% ethanol at 4°C before use. Scans were performed using approximately 15-μm voxel sizes (Phoenix nanotom™, GE measurement and control). The scanner used a tungsten source X-ray tube operating at 100 KV and 70 μA. Data acquisition from each sample required 90 minutes. Images were reconstructed using Phoenix datos*|x* CT software™. For measurements, the Microview™ software was used and the calibration for mineral density in the scans was performed with the use of a phantom (hydroxyapatite). For each sample, two regions of interest (ROIs) were designed manually corresponding to the subchondral bone area (less than 2 mm from the cartilage area) and to the trabecular area (between 3 and 4 mm under the cartilage area). Morphometry parameters measured were: bone mineral density (BMD), bone volume fraction (BV/TV), trabecular thickness (Tb.Th), trabecular number (Tb.N), and trabecular spacing (Tb.Sp). A principal component analysis (dudi.pca) was done using R scripts and the ade4 package. Likewise, between-class correspondence analysis (dudi.bca) was performed to identify the most significant parameters differentiating OC(D)-affected and OC-free samples. Hierarchical clustering was performed using R (hclust), based on the 3 most significant parameters.

### Metabolomics

Synovial fluid was collected from tibiotarsal joints from OC-free and OC(D)-affected foals. For foals not presenting bilateral OC(D) lesions, synovial fluid was also collected from the contralateral OC-free joint. Samples were thawed at room temperature and 600 μL added to 5-mm NMR tubes prefilled with 100 μL deuterium oxide for field locking. The proton spectra were acquired at 500 MHz on a Bruker Advance III spectrometer, with the noesy1D sequence for water suppression. The temperature was 297 K. The time domain raw signals (free induction decays: FIDs) were collected on 32 K points for a spectral window of 6000 Hz and 64 transients. The FIDs were processed with the MestReNOVA software. The Fourier transform was performed with an exponential function producing a 0.3 Hz line broadening. Spectra were phased and the baseline correction was performed with three points at 0, 5, and 9 ppm. Each spectrum was calibrated using acetic acid signal at 1.92 ppm in blood spectra. The spectral region between 0 ppm and 9 ppm was divided into 9000 spectral regions of 0.001 ppm width called buckets. Each bucket is labeled with its median chemical shift value. The water region, between 4.6 and 5 ppm, was excluded. Considering the possible variation of the sample concentration, the spectra were normalized according to the “Probabilistic Quotient Normalization” method [19]. Statistical analyses consisted of a Principal Component Analysis (PCA) performed using the statistics SIMCA P + software (ver. 12, Umetrics AB, Umea, Sweden). The results are presented as a score plot constructed with the two principal components describing the spectra and the loading plots describing the importance of the variables (here, the buckets) to compute the principal components.

## Results

### Osteochondrosis lesion typology

**Figure 2:**
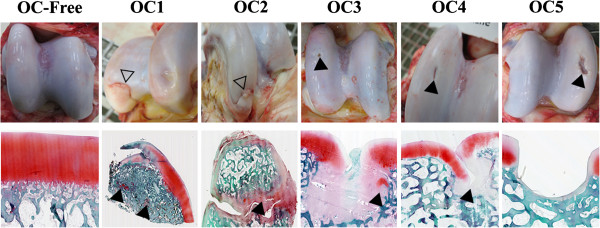
**Identification and characterization of OC(D) lesion subtypes.** Upper Panels - Macroscopic views of the *talus trochlea* of one OC-free and five OC-affected foals. Two major types of osteochondrosis lesions could be distinguished: cartilage fragments corresponding to OCD lesions (horses OC1, OC2, and OC4; open arrows) and osteochondral defect corresponding to OC lesions with varying degrees of cartilage loss (horses OC3, OC5, and OC4; black arrow). Lower Panels - Histological views of osteochondrosis lesions (light green/safranin-O stain). Irregularities and reduced thickness were observed at the cartilage surface. The absence of staining both in and close to the lesion indicates reduction in proteoglycan content. Abnormal cartilage cores were also seen in the subchondral bone (black arrows).

During necropsy, two major types of macroscopic lesions were observed on the lateral trochlear ridge of the talus of the five 10 month-old OC(D)-affected horses. Firstly, articular cartilage fragments were observed at the distal aspect of the trochlea, a typical location of OCD fragments. These samples were called OCD samples (horses OC1, OC2 on Figure 2, upper panel) and showed a fragment partially detached from the underlying subchondral bone. Secondly, some lesions located on the middle of the trochlea, resembled small osteochondral defects or holes in the cartilage, ranging from a minor superficial cartilage defect to an absence of articular cartilage as far as the subchondral bone (horses OC3, OC4, and OC5 on Figure 2). These samples were called “OC samples”. It is worth noting that OC4 presented both OCD and OC lesions. At necropsy, the subchondral bone of OC-affected horses seemed easier to cut.

Comparison between samples collected from the lateral trochlear ridge of the *talus* of OC-free horses and OC(D)-affected horses showed the presence of irregularities and reduced thickness of articular cartilage, especially for horse OC5 (lower panel Figure 2). Staining was absent for this animal, suggesting altered proteoglycan content. Horses OC1 and OC2 presented abnormal cartilage cores retained in the subchondral bone.

**Figure 3:**
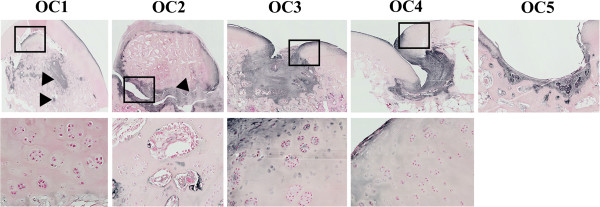
**Investigation of type-VI collagen localization and chondrocyte organization.** Upper Panel - Type-VI collagen localization by immunostaining. Both lesion types (OCD [*f*] and osteochondral defects (OC) [*s*]) were characterized by positively stained areas (arrows) indicative of cartilage dedifferentiation leading to the formation of local scar tissue (fibrocartilage). Lower Panel - Magnification views (x20) of animals OC1-OC4 showing chondrocyte clusters located surrounding ***(*** ***s*** ***)*** or close to the lesions ***(*** ***c*** ***)***, which may reflect a healing process is underway.

Type-I collagen immunostaining revealed large areas of chondrocyte dedifferentiation in the lesion. In addition, type-VI collagen was widely distributed in the lesion (upper panel Figure 3), contrasting with the usual localization at the pericellular region (chondron) in OC-free articular cartilage. Chondrocyte dedifferentiation and type-VI collagen expression are suggestive of fibrocartilage. Abnormal chondrocyte clusters were observed at different localizations: surrounding the lesions, close to the lesions, and distant from the lesions in horses OC1-OC4 (lower panel Figure 3). Based on histology, OC lesions were classified into two groups: 1) osteochondral defects with invagination of articular cartilage, progressive dedifferentiation and high type-VI collagen expression (horses OC3 and OC4); and 2) lesions with major cartilage loss (horse OC5), with low type-VI collagen expression.

### Proteomics comparative analysis

Comparison between bone and cartilage samples from the five OC-free (control samples) and the five OC(D)-affected horses (predisposed samples) was performed using our previously published method [18]. A total of 554 and 639 reproducible spots were observed on cartilage and bone gels, respectively.

**Figure 4:**
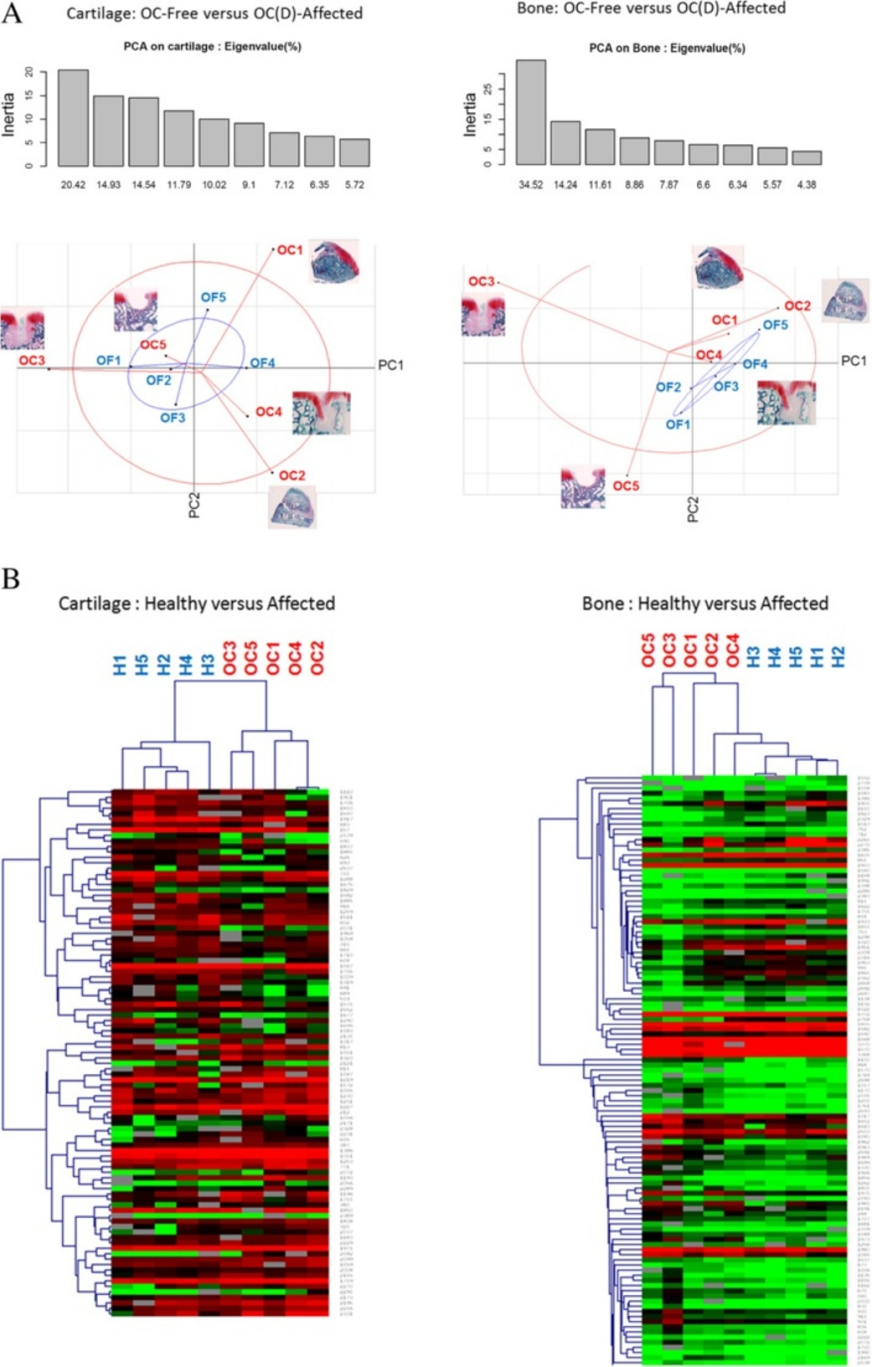
**Statistical distinction between OC-free (n = 5) and OC(D)-predisposed (n = 5) bone and cartilage samples. (A)** *Principal Component Analysis (PCA) of Proteomic Data*. The PCA reveals a great heterogeneity between predisposed samples in both cartilage and bone. Furthermore, OCD samples (horses OC1, OC2 and OC4) cluster near healthy samples in bone, in contrast to OC (osteochondral defects) (horses OC3 and OC5). This suggests that OCD may result mainly from a cartilage defect, whereas OC may result from a combined cartilage and bone defect. **(B)** *Hierarchical Clustering (HCL) of Proteomic Data*. Differentially expressed spots (97 in cartilage, 126 in bone) between healthy OC-free (n = 5) and predisposed (n = 5) samples collected from OC-affected and OC-free foals were used to perform HCL. A distinction could be made between OC-affected and OC-free foals, as well as OCD and OC (osteochondral defects) in both cartilage and bone. In addition, OCD-affected foals have similar profile to OC-free foals based on their bone proteome, suggesting that this lesion subtype shows only a minor defect in bone.

Principal Component Analysis (PCA) showed an important heterogeneity for cartilage and bone samples, especially for OC(D)-affected foals (Figure 4a). Hierarchical clustering (HCL) was also performed based on differentially expressed spots (Figure 4b). Histology, PCA and HCL corroborated the classification of samples as OCD and OC in both cartilage and bone. Furthermore, OCD-predisposed samples (horses OC1, OC2, and OC4) were clustered near OC-free samples in bone, in contrast to OC-predisposed ones (horses OC3 and OC5).

Between-class correspondence analysis highlighted 23 (cartilage) and 37 (bone) modulated proteins between OC-free and predisposed samples (Additional file 1: Tables S1 and S2). In cartilage, the identified differentially expressed proteins are mainly involved in growth, proliferation and development, cellular assembly and organization, cell communication, cell adhesion, lipid and protein metabolism, energy production, vesicle-mediated transport and immune system processes (Additional file 1, cartilage annotation). Proteins associated with osteoarticular diseases and developmental disorders such as achondrogenesis, dysplasia, or Stickler and Kniest syndromes were also modulated.

In subchondral bone, differentially modulated proteins are mainly involved in developmental processes, cellular component organization, cell-to-cell signaling, cell adhesion, protein metabolic processes, protein transport and response to stimulus (Additional file 1, bone annotation). Several plasma proteins, including fibrinogen-β and -γ, hemopexin, transferrin, complement component 4 binding protein α, as well as apolipoprotein A1 and fetuin B, were modulated in both OC- and OCD-predisposed samples. In addition, five of the modulated proteins had also been identified in synovial fluid samples from OC(D)-affected foals [20]: actin gamma 1, albumin, haptoglobin, fibrinogen beta chain and complement component 4 binding protein alpha (denoted by # in Additional file 1: Table S1 and Table S2).

### Validation by comparative μCT morphometry analysis of subchondral bone

**Figure 5:**
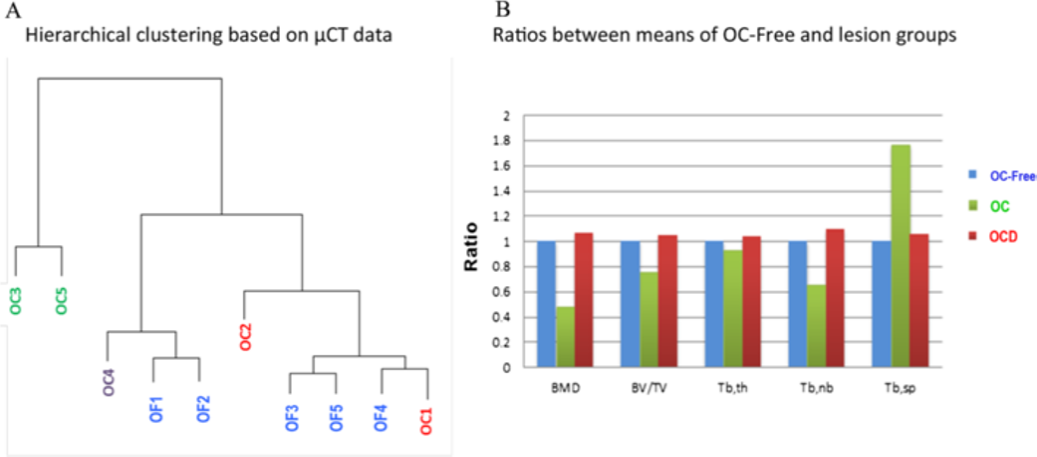
**Comparison of subchondral bone structure using micro computed tomography (μCT).** μCT analyses were performed to compare the subchondral bone structure between healthy (n = 5) and predisposed samples (n = 5). Five parameters were measured: the bone mineral density (BMD), the ratio between bone volume and total volume (BV/TV), the trabecular thickness (Tb,th), the trabecular number (Tb.nb) and the trabecular spacing (Tb,sp). Between-class correspondence analysis highlighted BMD, Tb,nb and Tb.sp as discriminating parameters between OC-affected and OC-free samples. Hierarchical clustering **(A)** based on these parameters showed that OCD-predisposed samples (horses OC1, OC2 and OC4) were clustered with healthy samples, whereas OC-predisposed samples (OC3 and OC5) were clustered separately. These observations suggested that OC-predisposed samples presented modified bone structure. The measures of the ratios between means of healthy and each predisposed group **(B)** seemed to confirm this assumption. The data suggested that the BMD and the Tb,nb were reduced in subchondral bone predisposed samples from osteochondral defect-affected foals, whereas Tb.sp was increased. Interestingly, the μCt analysis did not reveal any variation in bone-predisposed samples from OCD-affected foals.

Bone mineral density (BMD), bone volume fraction (BV/TV), trabecular thickness (Tb.Th), trabecular number (Tb.N), and trabecular spacing (Tb.Sp) were measured on subchondral bone from the middle of femoral trochlea explants. These parameters were used for BCA analysis, highlighting BMD, Tb.N and Tb.Th as discriminating parameters. Hierarchical clustering based on these parameters distinguished OC- from OCD-predisposed samples and OC-free samples, in agreement with observed histological features and data obtained from proteomics. Again, OCD-predisposed samples (horses OC1, OC2 and OC4) were clustered with OC-free samples (Figure 5 and Additional file 1, microCt).

Bone mineral density was significantly reduced in subchondral bone from OC-predisposed samples (horses OC3, OC4 and OC5; Figure 5). In addition, bone structure was disrupted, with samples showing a reduced number of *trabeculae* (30%) and increased trabecular spacing (80% increase). The μCt analysis did not reveal any variation in bone samples from foals affected by OCD.

### Metabolomic study of synovial fluid

**Figure 6:**
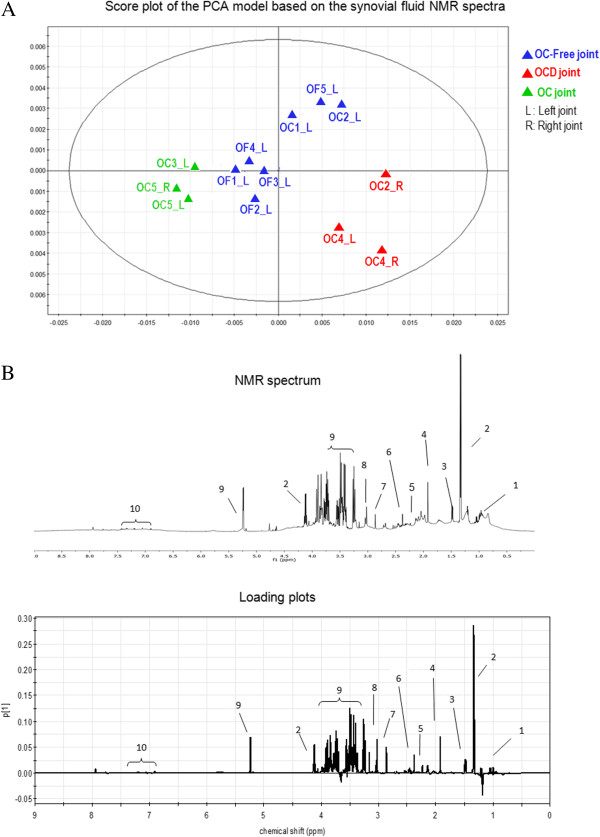
**Metabolomic study of synovial fluid.** Synovial fluids were collected from tibiotarsal joints from OC-free and OC-affected foals. When possible, synovial fluid from OC foals was also collected from the contralateral healthy joint. Synovial fluids were analyzed by NMR metabolomics and a PCA based on obtained spectra was performed **(A)** The score Plot of the PCA Model shows discrimination between healthy, OCD-affected and OC-affected joints, but not between healthy joints from OC-affected (OC1_L and OC2_L) and OC-free foals. L = left joint; R = right joint; OF = OC-free foal; OC = affected foal; Blue: healthy joint; Green: OC lesion (osteochondral defects); Red: OCD lesion (fragments). **(B)** Proton NMR spectrum of synovial fluid (upper graph) and loading plot (lower graph) of the first principal components obtained with the PCA model. The importance of the buckets (y-axis) is plotted as a function of their chemical shift (x-axis). The corresponding metabolites are increasing along the component when positive and decreasing along the component when negative. Metabolite attributions: 1: branched-chain amino acids including valine, leucine, and isoleucine; 2: lactic acid; 3: alanine; 4: acetic acid; 5: acetone; 6: pyruvic acid; 7: trimethylamine; 8: creatine; 9: glucose; 10: aromatic amino acids including tyrosine and phenylalanine.

A PCA based on the synovial fluid NMR spectra discriminated between OC-affected, OC-free and OCD-affected samples in PC1 (Figure 6A), in good agreement with proteomic and histological features. PC2 mainly discriminated injured and healthy joints from OCD-affected horses, but did not discriminate well between healthy joints from OCD-affected (OC1 and OC2 left joints) and OC-free horses. The loading plots (Figure 6B) provide the weight of each spectral region, showing that most metabolites positively contribute to PC1, with lactate, glucose, acetate, creatine and pyruvate having the heaviest loadings. Thus, elevated levels of these metabolites characterize injured joints from OCD-affected horses (OC2_R, OC4_L and OC4_R), and to some extent, healthy joints from OCD-affected horses (OC1_L and OC2_L). Elevated levels of lactate and acetate are suggestive of hypoxic and acidotic status whereas increased glucose and pyruvate levels indicate a low efficiency of glycolysis and reduced aerobic processes. Elevated levels of branched-chain amino acids and creatine may be indicative of increased proteolysis. In contrast, synovial fluid collected from joints presenting OC lesions (horses OC3_L, OC5_R and OC5_L) had reduced levels of glucose, pyruvate and lactate, and an increase in ketone bodies including hydroxybutyrate. This is suggestive of a reduced anaerobic glycolytic rate as well as the use of fatty acids as a source of energy.

## Discussion and conclusion

The terms “*osteochondrosis*” and “*osteochondritis dissecans*” have been overused in horses, being applied indiscriminately to a variety of lesions in the skeleton of horses, without respect to recognized differences in their etiology, pathogenesis and clinical characteristics. Conversely in humans, several severe conditions were initially grouped under the heading of *osteochondrosis* [21], but have now been subdivided into about 50 syndromes [22]. Based on a comprehensive and integrative approach including radiographic, histological, μCT, proteomic and metabolomic techniques, our study aimed at improving our understanding of equine osteochondrosis OC(D) physiopathology. Histology showed many features consistent with previously described OC(D) lesions in several species, including cartilage retained in the underlying subchondral bone, changes in matrix composition, chondrocyte clusters, and the presence of scar tissue similar to fibrocartilage [12]. Chondrocyte clusters have been described as a characteristic feature of osteoarthritic lesions and are thought to result from proliferating chondrocytes attempting to repair the tissue [23, 24]. In addition, lesions are commonly classified into fragments (OCD) and OC lesions (including cyst). Histology, PCA and HCL concurred with this commonly accepted classification. Histological features of samples from foals OC3 and OC4, showing a progressive invagination of cartilage into bone, suggest that these osteochondral defects may be early cystic lesions. However, it seems unlikely that the lesion observed for foal OC5 would evolve into a cyst since we observed a severe cartilage loss. Thus, histology revealed the heterogeneous nature of OC type lesions, showing various degrees of cartilage invagination and cartilage loss up to the subchondral bone. This heterogeneity may be related to a progressive degeneration of epiphyseal cartilage, leading finally to cartilage loss and development of a thin fibrocartilage layer, or alternatively, to different pathological entities.

Our proteomic study was designed to explore the hypothesis of a putative constitutive defect in epiphyseal cartilage and/or subchondral bone associated with the development of primary lesions. We focused on lesion-free cartilage and bone sampled from femoropatellar joints of OC-free and OC(D)-affected horses. Since OC(D) is a very dynamic disease, the reliable identification of OC-free controls is crucial and errors in foal classification may represent one potential weakness of this design. Therefore, radiographs and clinical examinations were performed on foals between six to seven months of age, when lesion number reaches a peak [17]. At necropsy (10 months of age, when lesions are well established and will persist thereafter), the presence of lesions was macroscopically checked for all forelimb and hindlimb joints, but cervical intervertebral joints, which could show a high incidence of OC, were not examined. Since limb and cervical lesion scores have been reported to be highly correlated (for instance 0.69 in [25]), errors in foal classification may however be limited. In addition, OC-free foals may be genetically predisposed but have not yet encountered environmental factors triggering the disease and we cannot rule out that OC-free foals may have had lesions which may have spontaneously healed before the first radiographic exam. These limitations apply to most studies performed to date on OC(D), especially genetic mapping studies. Furthermore, considering foals with spontaneous healing as OC(D) affected is still a subject of debates. Indeed, one could hypothesize that minor defects resembling dyschondroplasic lesions may be quite a common feature of developing cartilage, that resorb spontaneously except in OC(D) predisposed foals. In our opinion, our proteomic data provide good validation of the OC-free status, since control samples are well-clustered whereas affected samples exhibit a great variability as shown in our PCA analysis.

Our study adopted a novel integrative approach using several techniques to confirm observations, as opposed to a single technique approach which may have provided equivocal results. The weakness of the study lies with the animal species (horse) used which meant low n-numbers. The study was not designed to test any potential candidates. This could be the subject of another study. Based on our proteomic results, PCA showed that equine osteochondrosis predisposition might be due to a defect in both cartilage and bone tissues. Several proteins were found to be modulated and five of the differentially expressed proteins, ACTG1, ALB, HP, FBG and C4BPA (Additional file 1: Table S1), have also previously been identified in synovial fluid from OC(D)-affected horses [20]. In addition, a between-class correspondence analysis highlighted several proteins specifically involved in susceptibility to each lesion subtype, suggesting that common as well as distinct molecular mechanisms may be involved in the etiopathogenesis of each osteochondrosis subtype. This is in good agreement with the observed low phenotypic and genetic correlations between different joints [26–29], suggesting that OC and OCD may be considered different traits and that future genome-analysis studies should focus on predilection sites rather than the entire disease. Altogether, these data agree with the hypothesis that OC(D) joint specificity is due to the interaction of different molecular mechanisms, which are altered in all joints (systemic defect) but trigger a different susceptibility (joint specificity due to the different biomechanical constraints sustained by the joints).

### Molecular mechanisms in cartilage

In cartilage, the down-regulation of COL3A1 and up-regulation of COL6A1 and HAPLN1, proteins involved in ECM structure and chondrocyte microenvironment and micromechanical regulation [30] suggest a production of altered ECM. In addition, down-regulation of CLU, a glycoprotein providing a protective effect for cells at the fluid-tissue interface, is in line with the previously observed down-regulation in advanced osteoarthritic cartilage [31] suggesting a reduced protection against biomechanical stress.

Furthermore, the down-regulation of CKB and NDUFS1 may be indicative of an altered energy homeostasis leading to defective cartilage maturation in OC(D)-affected foals. CKB is required for normal development and mineralization of the growth plate and its expression level is related to chondrocyte maturation stages [32]. CKB is thought to play a key role during the switch from oxidative metabolism to glycolysis associated with the hypertrophic differentiation process. In line with a defective cartilage maturation hypothesis, up-regulation of proteins involved in vesicle transport and ubiquitin-dependent protein degradation by the proteasome (VCP and PSMB3), down-regulation of the chaperone proteins FKBP10 and CLU as well as translation elongation factor EEF1D suggest impaired protein biosynthesis, folding, secretion and degradation. Taken together, these findings are consistent with data obtained in human OCD fragments, showing chondrocytes with abnormal accumulation of ECM proteins in distended rough endoplasmic reticulum (ER) [33], suggesting that OCD might be an ER storage disease. However, some specificity was observed according to lesion subtype. For example in OC-predisposed cartilage, up-regulation of HSPA1A and PDIA3—which play a role in the folding of newly synthesized glycoproteins— suggests that a partial ER-stress response may have been triggered. Since ER stress triggers metabolism changes [34], such a hypothesis may be consistent with our metabolomic data, showing reduced glycolysis and increased utilization of fatty acids to produce energy, a characteristic feature of the inflamed rheumatoid joint [35]. Interestingly, our study in French trotters also identified ER stress and mitochondrial dysfunction as key factors involved in OCD [36]. In contrast, HSPA1A was down-regulated in OCD predisposed cartilage. However, in this latter case, modulation of additional genes involved in energy metabolism, such as ENO1 is consistent with our metabolomics findings, showing a reduced aerobic glycolysis and an increased proteolysis to overcome this defect and produce the required ATP.

These differences may lead to an alternative outcome according to lesion subtype: OC susceptibility appeared to be mainly associated with disrupted ECM structure. Down-regulation of several major ECM constituents such as COL2A1, COL3A1, SPARC and COL11A2, as well as COL11A1, which are essential for maintaining the spacing and diameter of type-II collagen fibrils [37] and MFG-E8, a protein preserving the structure of articular cartilage and stabilizing chondrocyte adhesion to the ECM [38], may be indicative of a defective ECM.

In contrast, OCD susceptibility seemed to be associated with impaired ECM mineralization, as suggested by down-regulation of AHSG, a glycoprotein which knockout in mice leads to growth plate defects, including a reduced number of hypertrophic chondrocytes as well as accelerated mineralization [39]. This defect may be caused by defective hypertrophic chondrocyte terminal differentiation and up-regulation of major cytoskeletal components (VIM, ACTG1, ACTA1). This is in agreement with the essential role of the cytoskeletal network during chondrogenesis and hypertrophy [40]. The defective cartilage mineralization occurring in OC may result in a failure of osteoclast remodeling. Decreased or delayed mineralization combined with altered ECM structure may therefore lead to impaired epiphyseal cartilage biomechanical properties, making it prone to the development of fragments.

### Molecular mechanisms in bone

Horses presenting OC lesions had softer and more vascularized subchondral bone, and osteonectin—a glycoprotein known to promote angiogenesis, increase metalloproteinase activity, and inhibit bone mineralization—was shown to be up-regulated, together with other plasma proteins. One of these was apolipoprotein A1, which in bone, may promote cholesterol efflux and affect HDL levels, leading to the alteration of bone mineral density and trabecular architecture. There is growing evidence suggesting that abnormalities in lipid metabolism may impact bone metabolism and mineral density. In particular, exogenous sources of cholesterol are essential for osteoclast formation as well as survival [41] and several studies have suggested a relationship between HDL, osteoblasts, osteoclasts, and bone mineral density (for review see [42]). Up regulation of AZGP1 - an adipokine involved in lipid metabolism - may provide clues about the link between body weight, conformation and OC susceptibility. Indeed a genetic linkage between AZGP1 and body weight has been shown in mice [43] and polymorphisms in this gene have been associated with high body weight, body length and chest girth in cattle [44].

OC-affected samples showed more modulated proteins than OCD-affected samples and fold changes were higher in these samples suggesting that OC lesions may result from a major bone defect, whereas OCD lesions may be associated with a slight bone defect. In line with our μCt data, defective mineralization and an altered subchondral bone structure are the main hallmarks of samples from OC-affected horses. Proteomic data agree with this finding since several modulated proteins are associated with bone mineralization and bone remodeling. For example, down-regulation of ATP6V1A may impair ATP production, which is essential for osteoclasts to generate the acidic environment required for the digestion of the organic bone matrix [45] as well as proper osteoblast differentiation and activation [46]. Likewise, down-regulation of CKB, AHSG and CA may also be associated with impaired bone mineralization process [47, 48].

Together, our results suggest that, in equine osteochondrosis, the different lesion subtypes may be due to different underlying molecular mechanisms and that the predisposition could be due to a defect in both bone and cartilage tissues. This is in line with observations made for osteoarticular diseases in other species, which are often associated with cartilage disruption extending into the underlying subchondral bone (for review see [49]). A constitutive cartilage defect may be responsible for OCD physiopathology, whereas combined cartilage and bone defects may trigger OC. However, the precise mechanisms linking cartilage and bone defects remain to be elucidated and it is not clear whether a primary cartilage defect triggers a bone defect (or *vice versa*) or whether both tissues are independently affected.

Differentially expressed plasma proteins highlighted in this study, such as haptoglogin, hemopexin, fetuin, apolipoprotein A1 and transferrin may represent relevant diagnostic biomarkers, but further studies are needed to evaluate their diagnostic or prognostic significance. Likewise, proton NMR spectroscopy enabled the investigation of low molecular weight molecules present in the synovial fluid from the tibiotarsal joints of OC-free and OC(D)-affected foals, a technique which has already been successfully applied to study pathological synovial fluids in humans [50] and horses [51]. In conclusion, our data will contribute to refining the definition of equine osteochondrosis lesion subtypes and the understanding of the underlying molecular mechanisms involved for their development. This may help improving diagnosis and developing relevant prevention strategies or treatments for horses and other species.

## Electronic supplementary material

Additional file 1: Table S1: Differentially expressed proteins in cartilage. **Table S2.** Differentially expressed proteins in bone. **Table S3.** List of foals examined to be included in the study. **S2 Cartilage annotations.** GO annotation of differentially expressed proteins in cartilage (list and pie chart); **S3 Bone annotations.** GO annotation of differentially expressed proteins in bone (list and pie chart); **S4 microCT.** Results of μCt studies; **Picture P1.** localization of the explants collected on the femoral trochlea for each foal. (XLS 316 KB)

## Abbreviations

OC: Osteochondrosis
OCD: Osteochondritis dissecans
ECM: Extra-cellular matrix
μCT: Micro computed tomography.
